# Endovascular Treatment for Acute Thromboembolic Occlusion of the Superior Mesenteric Artery and the Outcome Comparison between Endovascular and Open Surgical Treatments: A Retrospective Study

**DOI:** 10.1155/2017/1964765

**Published:** 2017-10-24

**Authors:** Zhao Zhang, Dan Wang, Guoxun Li, Ximo Wang, Yuxiang Wang, Gang Li, Tao Jiang

**Affiliations:** ^1^Department of Colorectal Surgery, Tianjin Union Medical Center, Tianjin 300121, China; ^2^Department of Pathology, Tianjin Medical University General Hospital, Tianjin 300000, China; ^3^Department of General Surgery, Tianjin Union Medical Center, Tianjin 300121, China; ^4^Department of General Surgery, Nankai Hospital of Tianjin, Tianjin 300000, China

## Abstract

We reported our experience with endovascular treatment for patients with acute thromboembolic occlusion of the superior mesenteric artery (ATOS) as well as comparing the efficacy between endovascular and traditional open surgical treatments. Eighteen consecutive patients with ATOS who received endovascular treatment and 12 patients who received open surgical treatment between February 2007 and October 2012 at Tianjin Union Medical Center (Tianjin, China) were retrospectively reviewed. Primary clinical outcomes included the technical success, requirement of laparotomy, length of bowel resection, perioperative mortality within 30 days, and surgical complications. The patients were followed up for 0.1 to 98 months. For patients who underwent endovascular treatment, complete technical success was achieved in 8 (44.4%) patients and partial success was achieved in the remaining 10 (55.6%) patients. Laparotomy was required in 6 (33.3%) patients. The 30-day mortality was 16.7%. In comparison to open surgical therapy, endovascular therapy achieved lower requirement of laparotomy (in 33.3% versus in 58.3% of cases, *p* = 0.18), significantly shorter average length of bowel resection (88 ± 44 versus 253 ± 103 cm, *p* = 0.01), and lower mortality rate (16.7% versus 33.3%, *p* = 0.68). The endovascular therapy is a promising treatment alternative for ATOS.

## 1. Introduction

Acute thromboembolic occlusion of the superior mesenteric artery (ATOS) is a life-threatening disease characterized by strangulated obstruction and a final physiopathology of bowel necrosis and poor patient prognosis. Despite improvement in living standards, the incidence of ATOS has gradually risen in recent years. The mortality rate of ATOS has remained fairly high (ranging from 60% to 90%) over the past decades [1–5], which is at least partly attributable to its unspecific symptoms and lack of reliable examinations. Surgery for ATOS is associated with a high rate of morbidity and mortality [6, 7]. Over the last years, a few studies reported the employment of endovascular revascularization in the treatment of ATOS [8–12], with rapid blood supply reestablished and relatively small resultant trauma. It was shown by very few studies that endovascular treatment resulted in significantly less bowel resection and shorter bowel syndrome and mortality than open surgery [13]; however, whether or not endovascular interventional therapy should be the primary treatment for ATOS is still controversial [14, 15].

In the present study, we retrospectively analyzed the clinical data from cases of 30 patients with ATOS admitted to the Tianjin Union Medical Center (Tianjin, China) in an effort to assess the efficacy of endovascular therapy for ATOS as well as to compare the outcomes between endovascular and open surgical treatments.

## 2. Materials and Methods

### 2.1. Patients

**Figure 1:**
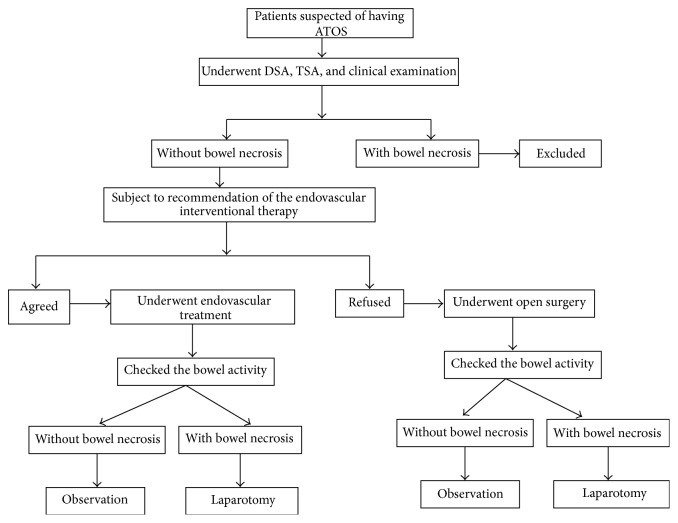
The flow chart of treatments for patients with ATOS.

This study was approved by the Institutional Review Board of Tianjin Union Medical Center (Tianjin, China). Cases of arterial thrombosis admitted to the Tianjin Union Medical Center from February 2007 to October 2012 were retrospectively reviewed. Patients with ATOS confirmed by computed tomography angiography (CTA) or digital subtraction angiography (DSA) examination were included. Patients with symptoms (e.g., abdominal peritonitis and systemic toxic symptoms) of advanced bowel ischemia or bowel necrosis evidenced by CTA observations (e.g., with air bubble sign but without intestinal wall contrast enhancement on CTA images) were excluded. Before the treatment, the patients and their relatives were informed and the endovascular interventional therapy was recommended. If agreed, they received endovascular interventional therapy; but if they refused, conservative anticoagulant therapy would be carried out (Figure 1). Patient demographics, clinical information, and procedural data were gathered from the medical records.

According to the initial treatment program, cases were classified into an endovascular treatment group (endovascular group) and a traditional open surgical treatment group (open surgery group). Endovascular therapy included aspiration, thrombolysis, anticoagulation, balloon dilatation, stent implantation, and surgical intervention. Open surgical therapy included anticoagulant and thrombolytic treatment, laparotomy, excision of the necrotic bowel, arterial embolectomy, intestinal fistulation, and delayed abdominal closure. The time from symptom onset to treatment was recorded on the basis of the duration of pain experienced by the patient and the included diagnostic evaluation before treatment. The time of surgical intervention was also recorded.

### 2.2. Endovascular Treatment

All procedures were performed in a surgical theatre equipped as an Angiography Suite. Femoral access was routinely gained using a 6 F introducer (Launcher Guiding Catheter, Medtronic Inc., Danvers, MA, USA), with brachial access where necessary. A bolus of 3–5,000 units of heparin was immediately administered through the introducer, followed by 1,000 units/hour of heparin administration throughout the procedure. Activated clotting time was controlled around 200 seconds. Arterial anatomy was established by abdominal aortogram in both the anterior-posterior and lateral views. Once the superior mesenteric artery (SMA) was confirmed, the 6 F introducer was replaced with an 8 F introducer (Launcher Guiding Catheter). An 8 F guiding catheter (Launcher Guiding Catheter) was advanced through the 8 F introducer and the guide wire was placed beyond the mesenteric occlusion. A 6 F guiding catheter (Launcher Guiding Catheter) was passed through the 8 F guiding catheter and also placed beyond the occlusion. Thrombus aspiration was performed through the 6 F guiding catheter (which was simultaneously and gradually withdrawn) with a 50 mL syringe. In cases where the occlusion was in the SMA branches, a 5 F catheter (Beacon Tip Torcon NB Advantage Catheter, Cook Co. Ltd., Bloomington, IN, USA) was used. Catheter directed thrombolysis and balloon catheter dilation were applied in operation. Retaining the catheter in the lesion, thrombolysis of urokinase (Livzon Pharmaceutical Group Inc., Zhuhai, China) (e.g., 200–400,000 units), vasodilation of papaverine (Shandong Hualu Pharmaceutical Co. Ltd., Chiping, China), and anticoagulation of heparin were applied locally. Particularly, urokinase in saline or 5% glucose solution was initially intravenously administered at a dose of 4,400 U/kg and a rate of 90 ml/h for 10 min, followed by intravenous administration at a rate of 4,400 U/h continuously for 2 h or 12 h. If residual branching vascular occlusion existed, microcatheters were placed in the arterial branches (i.e., ileocolic artery, ileum artery, and right colonic artery) for continued treatment. In the absence of stenosis of the mesenteric arterial trunk and branch, balloon dilation was performed for partial thrombi in order to force the thrombus towards the opposite side of the blood vessel. An adjunctive stent was placed when residual luminal narrowing was greater than 75% as a result of underlying atherosclerosis.

Technical success of the endovascular revascularization procedure was determined as the residual stenosis of the formerly occluded artery less than 30% in diameter and without migration of small thromboemboli towards branches, along with rapid flow and visible contrast reaching the whole bowel. Partial success was determined as reestablished or improved flow of contrast to the corresponding bowel either with residual luminal caliber more than 30% or as occurrence of small thromboemboli migration towards distal vessels [16, 17].

The endovascular device (e.g., mesenteric intravascular transcatheter) was removed after 48–72 h, by which the compression time of the puncture point was expected to be sufficient. Systemic anticoagulation and thrombolysis treatments were required after surgical treatment. All patients were admitted to intensive care unit for monitoring of any potential worsening of mesenteric ischemia or other complications from the procedure. Laparotomy was determined by the patient's clinical status (if the patient felt more serious abdominal pain or developed new symptoms that suggest bowel perforation and/or gangrene), but this was ultimately determined by the surgical staff (vascular and colorectal surgeons). Bowel viability and length of bowel for resection was also determined by a colorectal surgeon.

Low molecular weight heparin (GlaxoSmithKline Co. Ltd., London, UK) was administered subcutaneously at a dosage of 0.1 mL/10 kg at 12-hour interval for a period of 5 days. Once the abdominal pain related to bowel ischemia was resolved, warfarin was administered. Patients who had received a stent were treated with antiplatelet therapy, including 75 mg/day clopidogrel (oral) for 3 months and 100 mg/day aspirin (oral) for at least 12 months. Mortality, especially all in-hospital deaths within 30 days (30-day mortality), was recorded.

### 2.3. Open Surgical Treatment

Systemic treatment of thrombolysis with anticoagulants and blood vessel dilation should be used for confirmed ATOS cases. Different surgical approaches were determined by the observed intraoperative conditions and included intestinal resection, arterial embolectomy, intestinal fistulation, and delayed abdominal closure. Thrombolysis treatments and anticoagulants should continue to be administered after surgical intervention. If intestinal necrosis occurred according to the observed intestinal activity and clinical manifestation, a second surgical procedure (laparotomy) should be performed for bowel resection. The rough procedure was shown in Table S1 in Supplementary Material available online at https://doi.org/10.1155/2017/1964765. After the operation, patients got anticoagulant therapy for half a year. Mortality, especially 30-day in-hospital mortality, was also recorded.

### 2.4. Postoperative Complications and Follow-Up

The patients were followed up through telephone calls and outpatient clinic visits for 0.1 to 98 months. Renal failure was determined as urine creatinine more than 1.5 mg/dL in patients with normal renal function or an increase over 20% in patients with chronic renal failure during the postoperative period. Respiratory failure was defined in cases of patients requiring intubation over 72 hours [18].

### 2.5. Statistical Analysis

Data was expressed as a proportion of the dichotomous variables, the mean ± standard deviation (SD) for parametric continuous variables, or the median and interquartile range (25th–75th percentiles) for nonparametric continuous variables. Data were analyzed using the Statistical Package for the Social Sciences software (SPSS version 17.0, SPSS Inc., Chicago, IL, USA). Comparison between the endovascular treatment and open surgical treatment groups was carried out using Student's *t*-test for parametric data and Mann–Whitney *U* test for nonparametric data. In addition, *χ*^2^ test was used for comparing nominal data (shown as proportions) and Fisher exact test was applied as appropriate. Data were considered statistically significant with a *p* value < 0.05.

## 3. Results

### 3.1. Treatment Efficacy of Endovascular Therapy

**Figure 2:**
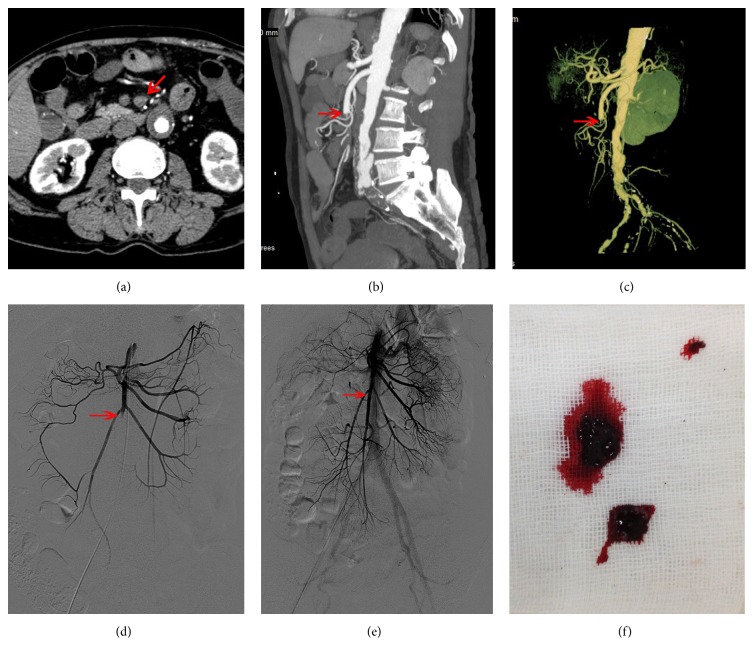
A 41-year-old female patient (Case (9) in Table 1) with acute thromboembolic occlusion of the superior mesenteric artery presented with acute-onset abdominal pain beginning 12 hours before the admission. ((a)–(c)) Computed tomography angiography of the abdomen indicated abrupt occlusion of the superior mesenteric artery (SMA) (arrow). (a) Cross-sectional image; (b) sagittal image; (c) three-dimensional lateral image; ((d), (e)) digital subtraction angiography (DSA) demonstrating the occlusion of the SMA before the treatment ((d), arrow) and complete restoration of the flow in SMA after the aspiration and thrombolysis ((e), arrow); (f) aspirated thromboemboli that were observed. Arrows indicate the morphological characteristics of artery.

Eighteen patients with ATOS received endovascular treatment, including 12 males and 6 females; mean age was 60.2 ± 10.9 (range 40–82) years. The characteristics, treatment, and outcomes of patients who received endovascular therapy were summarized in Table 1. Two patients had 75–90% occlusion, and the others had over 90% or complete occlusion. All patients exhibited abdominal pain and 2 patients had additional abdominal tenderness and rebound. Fourteen patients were found with main trunk occlusions (7 complete and 7 incomplete), 1 was found with branch occlusion (complete), and 3 were found with trunk occlusions mixed with branch occlusions (1 complete and 2 incomplete). No clinical or CT evidence of advanced bowel ischemia was observed. The interval from symptom onset to treatment was 20.8 ± 15.2 hours, more than 12 hours in 8 cases while less than 12 hours in 10 cases. All patients were treated for thrombolysis by use of a transcatheter. Nine (50%) of these patients received thrombus aspiration, 2 (11.1%) received balloon dilatation, and 1 (5.6%) required stent implantation. Complete technical success was achieved in 8 (44.4%) cases and partial success in the remaining 10 (55.6%) cases. A typical case of a 41-year-old female patient (Case  (9) in Table 1) with complete restoration of the flow in SMA after aspiration and thrombolysis was shown in Figure 2.

Despite the overall success observed in the endovascular therapy group, intestinal ischemic necrosis occurred in 6 cases after the endovascular treatment, which included the symptoms as follows: (1) abdominal puncture with bloody fluid, indicating serious intestinal ischemia; (2) serious gastrointestinal bleeding (including hematemesis and hematochezia), suggesting intestinal ischemia; (3) the phenomena of mucosa ischemia without reinforcement, intestinal wall pneumatosis, and intravascular pneumatosis in enhanced CT. All these indicated intestinal ischemic necrosis. These 6 patients had to undergo a laparotomy with an average resected bowel length of 88 ± 44 (20–150) cm^−1^ to treat new abdominal tenderness and rebound tenderness and 5 for worsening abdominal tenderness and rebound. Among these laparotomy-treated patients, 1 achieved complete technical success and the remaining 5 achieved partial success.

All the 2 patients with ATOS who had 75–90% occlusion achieved complete technique success. For the other 16 patients who had over 90% or even complete occlusion, 5 achieved complete technique success, and other 11 achieved partial technique success. Since the median interval from symptom onset to treatment of the patients was 12 (4–48) hours, the onset time of patients was classified as more than and within 12 hours, respectively. In 10 cases with the onset of symptoms to treatment time less than 12 hours, complete technical success was achieved in 6 (60%) cases and partial success in the remaining 4 (40%). Three patients further underwent a laparotomy, with the length of the resected bowel of 87 ± 23 (range 60–100) cm. In another 8 cases with the onset of syndrome to treatment time greater than 12 hours, complete technical success was achieved in 2 (25%) cases and partial success was achieved in the remaining 6 (75%). Three patients further underwent a laparotomy, with the length of the resected bowel of 90 ± 66 (range 20–150) cm.

Patients were followed up for 34.5 ± 27.1 (ranging from 0.1 to 98) months. Twelve (12/18, 66.7%) patients completely recovered from the symptoms, 2 (2/18, 11.1%) patients just felt occasional abdominal pain, and 4 (4/18, 22.2%) patients died (Table 1). Three (3/18, 16.7%) patients died within 30 days, including 1 (10.0%) of 10 patients with onset time within 12 h and 2 (25.0%) of 8 patients with onset time over 12 h. In addition, 1 patient died at 4 months. Among the 4 dead cases, 1 (12.5%, 1 of 8 patients) was with complete technical success and 3 (30%, 3 of 10 patients) with partial success. Causes of death included mesenteric ischemia-combined pyemia and multiple organ failure, gastrointestinal bleeding, myocardial infarction, and/or cardiac arrest.

### 3.2. Basic Clinical Data for Endovascular versus Open Surgical Treatment Groups

Eighteen patients who received endovascular treatment and 12 who received open surgical treatment were included in the present study. Totally, there were 19 male and 11 female patients. The mean age of the study population was 61.9 ± 11.1 years. There were no statistical differences between the 2 groups with regard to age, sex, comorbidity (including hypertension, diabetes mellitus, chronic renal failure, peripheral arterial disease, atrial fibrillation, and rheumatic heart disease), symptoms (including abdominal pain, nausea, intestinal obstruction, and bloody diarrhea), and laboratory results (including white blood cell count, blood urea nitrogen, creatinine, aspartate transaminase, alanine transaminase, lactate, D-dimer, and fibrinogen) (all *p* > 0.05, Table 2).

### 3.3. Operative Data for Endovascular versus Open Surgical Treatment Groups

As presented in Table 3, no statistical differences were observed between the 2 groups with regard to the time from onset of symptoms up to point of treatment (endovascular group 20.8 ± 15.2 versus open surgery group 25.8 ± 11.3 hours, *p* = 0.35) and the time between initial treatment and subsequent surgical exploration (endovascular group 26.3 ± 16.8 versus open surgery group 18.0 ± 7.7 hours, *p* = 0.26).

In endovascular treatment group, 6 (33.3%) of 18 patients required a laparotomy. In contrast, in open surgical treatment group, 7 (58.3%) of 12 patients required a laparotomy, with the average bowel resection of 253 ± 103 cm. Particularly, 2 cases in the open surgical treatment group required a second surgical procedure with 2 further cases resulting in fistula formation. Endovascular therapy required laparotomy in fewer cases in comparison with open surgical therapy (33.3% versus 58.3%, *p* = 0.26). The average length of bowel resection in the endovascular group was significantly less than in the open surgical group (88 ± 44 cm versus 253 ± 103 cm, *p* = 0.01).

### 3.4. Prognostic Data for Endovascular versus Open Surgical Treatment Groups

Patients in the open surgical treatment group were followed up for 13.9 ± 17.6 (range 1–48) months. Three (3/12, 25%) patients completely recovered from the symptoms, 2 (2/12, 16.7%) felt occasional abdominal pain, and 6 (6/12, 50%) died. Four (4/12, 33.3%) of them died within 30 days. Thirty-day mortality in the endovascular group (16.7%, 3 of 18 patients) was lower than in the open surgical treatment group (33.3%, 4 of 12 patients) (*p* = 0.68).

During the follow-up, there were 4 cases of respiratory failure (33.3%, 4 of 12) and 2 cases of renal failure (16.7%, 2 of 12) in the open surgical treatment group. In contrast, endovascular group had a lower rate of complications, with 3 cases (16.7%, 3 of 18 patients) of respiratory failure and 1 case of renal failure (5.6%, 1 of 18).

## 4. Discussion

Given the low incidence of ATOS, it is challenging for any treatment center to provide feedback on ATOS cases within a relatively short period. There have been a few related studies reporting small sample sizes [8–12, 19, 20]. However, whether or not interventional therapy should be the primary treatment for ATOS is still controversial [14, 15]. In this study, we reported the endovascular treatment outcomes of 18 patients with ATOS in China and showed that endovascular treatment exhibited advantages in the lower laparotomy requirement, shorter average length of bowel resection, and lower 30-day mortality compared with open surgical treatment.

The exclusion of intestinal necrosis is an important indicator for the use of endovascular treatment; however, the present study showed that technical success in reestablishing SMA flow might not necessarily prevent the occurrence of irreversible bowel ischemia. Therefore, any change in vital signs or abdominal conditions should be closely monitored following endovascular treatment. When accompanied by serious peritonitis and abdominal puncture with digestive juice leakage, intestinal necrosis is simple to diagnose: diagnosing early intestinal necrosis is far more challenging. Surgical laparotomy should be proactively performed as soon as intestinal necrosis is found and the choice of procedure should be based on the principle of “damage control.” A hybrid operating room should be set up in order to manage this as quickly as possible in failed endovascular cases.

Generally, endovascular revascularization has not been widely used for ATOS yet. Over the past years, there are a few reports demonstrating the treatment of ATOS through endovascular revascularization, mainly in case reports [8–12, 18–21]. Arthurs et al. showed endovascular treatment achieved technical success in 87% patients, with the requirement of laparotomy in 69% patients and mortality rate of 36% [18]. Puippe et al. reported that after the endovascular revascularization 38.5% patients obtained technical success with complete restoration, 38.5% required laparotomy, and 30.8% died within 30 days in hospital [12]. Raupach et al. revealed complete recanalization in 91.9% of patients who received endovascular management, with the requirement of laparotomy in 73.0% patients and in-hospital mortality of 27.0% [21]. Our study demonstrated that, for patients with ATOS who received endovascular therapy, 44.4% achieved complete technical success, 33.3% required laparotomy, and 30-day in-hospital mortality was 16.7%. The discrepancy of the reported treatment efficacy not only was related to the interval from onset of symptoms to intervention but also reflected the difference in the development of endovascular therapy for ATOS between countries. In China, there was only one case series study reporting early endovascular revascularization for 21 patients with ATOS [11]. Compared with Jia et al.'s study, we showed more requirement of laparotomy (6 of 18 cases, 33.3% versus 5 of 21 cases, 23.8%) and higher 30-day mortality (16.7% versus 9.5%). This may be attributable to the longer duration from symptom onset to treatment in our study (20.8 ± 15.2 versus 8.7 ± 4.1 hours). Nevertheless, the present study still showed more completely successful endovascular revascularization (44.4%, 8 of 18 cases, versus 33.3%, 6 of 21 cases).

The key to endovascular treatment is to establish blood flow quickly before irreversible intestinal necrosis occurs, in which the advantages of endovascular therapy lie. Block et al. showed less frequent bowel resection and lower 30-day mortality occurred in patients with ATOS by the endovascular treatment than by open surgical treatment [13], but these two types of treatments were not performed in the same hospitals. This means that study might not be able to correctly compare the treatment efficacies between endovascular and open surgical treatments. Arthurs et al. reported that, compared with traditional open revascularization therapy, endovascular treatment can result in lower laparotomy rates, a significantly decreased rate of bowel resection during surgical exploration, significantly lower in-hospital mortality, and fewer complications as a result of respiratory or renal failure [18]. However, that study just reviewed and analyzed the cases from 1999 to 2008 in USA, during which time the endovascular therapy had not been developed so well. In this study, we summarized our experience from February 2007 to October 2012 in China. Similarly, our study showed that endovascular treatment reduces the laparotomy rate when compared with open surgical treatment, although this is not statistically significant (33.33 versus 58.33, *p* = 0.26). In addition, the average length of bowel resection in the endovascular group was significantly less than in the open surgery group (88 ± 44 versus 253 ± 103 cm, *p* = 0.01). Thirty-day mortality in the endovascular group was lower than in the open surgery group (16.7% versus 33.3%, *p* = 0.68). Therefore, at least to some extent, early endovascular treatment can improve outcomes and reduce complications. Probably because of the small sample size, the significant advantage of endovascular treatment in decreasing of the requirement of laparotomy and mortality was not observed in this study, which needs further validation in the future study involving more patients.

In this study, the interval between the symptom onset and the admission was similar among the 2 groups. Since endovascular group patients received therapy concurrent with the diagnosis immediately after the admission while the open surgery group patients usually underwent anticoagulant therapy only after the CTA examination, the open surgical treatment appeared to be later than the endovascular treatment. This means that the actual interval from symptom onset to treatment between endovascular treatment and open surgical treatment groups was more similar than what appeared to be. This indicated that the better outcomes achieved by the endovascular therapy were not attributable to the difference of the interval from symptom onset to treatment between the two groups.

For patients with ATOS, the tolerance time of intestinal ischemia (without symptoms of advanced intestinal ischemia) is related to the duration of tissue endurance for ischemia, atherosclerosis, collateral circulation, and location of the obstruction. ATOS should be diagnosed and treated as early as possible and it was even recommended to have rapid diagnosis and treatment within 4 to 6 hours after the onset of symptom [22]. Longer delay before the treatment was related to mortality [23]. Revesz reported that more than 12 hours of intestinal ischemia contributed towards an increased mortality rate in patients with ATOS [5]. In the endovascular group in the present study, we observed more complete technical success (60%, 6 of 10 cases) and lower 30-day in-hospital mortality (10%, 1 of 10 cases) in cases with onset time less than 12 hours compared with those with onset time over 12 hours (complete technical success: 25%, 2 of 8 cases; 30-day mortality: 25%, 2 of 8 cases). The present result further suggests that the use of early endovascular therapy for ATOS in less than 12 hours from symptom onset is beneficial towards reducing mortality. Shorter time (e.g., <12 h) from symptoms onset to recanalization might be a predictor of good clinical outcome, which needs to be validated through multivariate analysis involving enough patient number.

This study has some limitations. This is a retrospective study that is not prospectively designed, which might bring about patient selection bias and incompleteness of some important information of patients. Furthermore, the sample size was relatively small, which might be the reason why the endovascular treatment did not exhibit significant advantages in decreasing the requirement of laparotomy, mortality, and so on over the nonendovascular treatment. In the next study, a prospective randomized controlled trial with more patients will be carried out to validate these results. In addition, in the future when more cases of patients with ATOS who receive endovascular treatment can be collected, the risk factors (probably including type of occlusion, time interval from onset to treatment, etc.) that influence the outcomes of endovascular treatment will be analyzed.

## 5. Conclusions

Endovascular treatment might be a promising treatment method for patients with ATOS, which improves patient outcomes and reduces complications. Further clinical and evidence-based researches are required.

## Supplementary Material

Table S1 Open surgical treatment.

## Figures and Tables

**Table 1:** Characteristics of patients who received endovascular therapy.

Patient number	Sex/age	Symptom	Location	Occlusion	Onset (hour)	Treatment	Technical success	TTL (hour)	LBR (cm)	Follow-up (month)	Outcome
(1)	M/82	AP, ATR	Trunk	>90%	12	Thrombolysis, laparotomy	Partial	6	100	1	Died

(2)	F/58	AP	Trunk	75%–90%	24	Aspiration, thrombolysis	Complete	No	No	48	Completely recovered

(3)	M/58	AP, ATR	Trunk	>90%	24	Thrombolysis, laparotomy	Partial	24	20	55	Completely recovered

(4)	M/66	AP	Trunk	Complete	8	Thrombolysis, laparotomy	Partial	48	100	48	Occasional AP

(5)	M/60	AP	Trunk	Complete	25	Thrombolysis	Partial	No	No	12	Occasional AP

(6)	F/72	AP	Trunk + branch	>90%	4	Aspiration, thrombolysis	Complete	No	No	34	Completely recovered

(7)	F/55	AP	Trunk + branch	Complete	48	Thrombolysis, laparotomy	Partial	36	100	0.1	Died

(8)	F/67	AP	Trunk	Complete	48	Thrombolysis	Partial	No	No	1	Completely recovered

(9)	F/41	AP	Trunk	>90%	12	Aspiration, thrombolysis	Complete	No	No	43	Completely recovered

(10)	M/46	AP	Branch	Complete	10	Aspiration, thrombolysis	Complete	No	No	98	Completely recovered

(11)	M/61	AP	Trunk	75%–90%	12	Aspiration, thrombolysis Balloon dilatation	Complete	No	No	41	Completely recovered

(12)	M/40	AP	Trunk + branch	>90%	40	Thrombolysis	Complete	No	No	4	Died

(13)	M/64	AP	Trunk	>90%	12	Aspiration, thrombolysis	Partial	No	No	36	Completely recovered

(14)	M/52	AP	Trunk	>90%	12	Aspiration, thrombolysis, stent implantation	Partial	No	No	52	Completely recovered

(15)	M/66	AP	Trunk	Complete	10	Aspiration, thrombolysis, laparotomy	Complete	36	60	36	Completely recovered

(16)	F/57	AP	Trunk	Complete	20	Thrombolysis, balloon dilatation	Partial	No	No	42	Completely recovered

(17)	M/62	AP	Trunk	Complete	6	Aspiration, thrombolysis	Complete	No	No	69	Completely recovered

(18)	M/75	AP	Trunk	Complete	48	Thrombolysis, laparotomy	Partial	8	150	1	Died

M: male; F: female; AP: abdominal pain; ATR: abdominal tenderness and rebound; LBR: length of bowel resection; TTL: time to laparotomy.

**Table 2:** Basic characteristics and perioperative biochemical parameters between endovascular and open surgical treatment groups.

Variable	All patients	Endovascular group	Open surgery	*p* ^a^	*t*
	(*n* = 30)	(*n* = 18)	group (*n* = 12)		
Age (year)	61.9 ± 11.1	60.2 ± 10.9	64.4 ± 11.5	0.32	1.01
Male (%/*n*)	63.3 (20)	66.7 (12)	58.30 (7)	0.71	
Etiology (%/*n*)				<0.05	
Embolic	70% (21)	83.3% (15)	50% (6)		
Thrombotic	30% (9)	16.7% (3)	50% (6)		
Comorbidity (%/*n*)					
Hypertension	50% (15)	33.3% (6)	75% (9)	0.06	
Diabetes mellitus	13.3% (4)	11.1% (2)	16.6% (2)	0.68	
Chronic renal failure	6.7% (2)	11.1% (2)	0	0.5	
Peripheral arterial disease	20% (6)	16.7% (3)	25% (3)	0.66	
Atrial fibrillation	53.33% (16)	50% (9)	58.33% (7)	0.72	
Rheumatic heart disease	20% (6)	27.78% (5)	8.33% (1)	0.36	
Active smoking	16.6% (5)	11.1% (2)	25% (3)	0.364	
Previous myocardial infarction	3.33% (1)	0	8.33% (1)	0.4	
History of thrombotic event	3.33% (1)	0	8.33% (1)	0.4	
Abdominal pain (%/*n*)	96.67% (29)	94.44% (17)	100% (12)	1.0	
Nausea (%/*n*)	76.7% (23)	88.89% (16)	58.3% (7)	0.08	
Emesis (%/*n*)	46.67% (14)	55.55% (10)	33.33% (4)	0.28	
Bloating (%/*n*)	60% (18)	61.11% (11)	58.33% (7)	1.0	
Bloody diarrhea (%/*n*)	40% (12)	55.55% (10)	16.67% (2)	0.06	
Intestinal obstruction (%/*n*)	20% (5)	11.11% (2)	25% (3)	0.36	
WBC count (10^3^/dL)	15.9 ± 4.8	14.9 ± 3.6	17.3 ± 6.1	0.198	1.3
Blood urea nitrogen (IQR) (mg/dL)	7.2 (5.3–8.7)	7.1 (5.7–8.6)	8.0 (5.0–8.8)	0.82	0.23
Creatinine (mg/dL)	75.7 ± 5.5	73.7 ± 7.1	78.7 ± 8.8	0.661	0.44
Potassium (mg/dL)	3.9 ± 0.1	3.9 ± 0.1	4.0 ± 0.2	0.49	0.7
pH	7.4 ± 0.01	7.4 ± 0.01	7.4 ± 0.01	0.512	0.67
Aspartate transaminase (IQR) (U/L)	30 (19.0–45.3)	32.5 (20–51)	28 (18.3–42.5)	0.785	0.28
Alanine transaminase (IQR) (U/L)	20.5 (15.5–44)	21.5 (18.5–4.8)	20 (11–39)	0.26	1.2
Lactate (IQR) (mmol/L)	2.6 (1.6–3.7)	2.0 (1.3–3.3)	3.0 (2.0–4.0)	0.347	0.96
Maxim lactate (IQR) (mmol/L)	2.6 (2.0–4.0)	2.3 (1.7–3.3)	3.5 (2.0–4.8)	0.125	1.6
D-dimer (mg/L)	5.1 ± 1.2	4.9 ± 1.5	5.4 ± 1.9	0.831	0.22
Fibrinogen (g/L)	4.3 ± 0.4	3.8 ± 0.3	5.2 ± 0.74	0.053	2.02

WBC, white blood cell; IQR, Interquartile range; a denotes comparisons between endovascular therapy and open surgical therapy.

**Table 3:** Therapeutic efficacy between endovascular and open surgery groups.

Variable	Endovascular group (*n* = 18)	Open surgery group (*n* = 12)	*p*	*t*
Symptom onset to treatment (h)	20.8 ± 15.2	25.8 ± 11.3	0.35	−0.96
Laparotomy required (%/*n*)	33.33 (6)	58.33 (7)	0.26	
Time to laparotomy (h)	26.3 ± 16.8	18.0 ± 7.7	0.26	1.18
Bowel resection (cm)	88 ± 44	253 ± 103	**0.01**	3.85
Thirty-day mortality (%/*n*)	16.7 (3)	33.3 (4)	0.68	
