# Supplementary material for: Endovascular Treatment for Acute Thromboembolic Occlusion of the Superior Mesenteric Artery and the Outcome Comparison between Endovascular and Open Surgical Treatments: A Retrospective Study
Source: Biomed Res Int. 2017 Oct 24;2017:1964765. doi: 10.1155/2017/1964765 (PMC5674482; doi:10.1155/2017/1964765)
Supplement: Supplementary file 1 — Table S1 Open surgical treatment. [file 1964765.f1.pdf]

Table S1 Open surgical treatment

Treatment means	Treatment time
Thrombolysis with anticoagulants and blood vessel dilation	Used after the confirmation of ATOS
Intestinal resection	Surgeries determined by the observed intra-operative conditions
Arterial embolectomy	
Intestinal fistulation	
Delayed abdominal closure	
Systemic anticoagulation and thrombolysis	After surgical intervention
A second surgical procedure	If intestinal necrosis occurred
